# The association between diverse serum folate with MAFLD and liver fibrosis based on NHANES 2017–2020

**DOI:** 10.3389/fnut.2024.1366843

**Published:** 2024-03-19

**Authors:** Jiacheng Cai, Dahua Chen, Wenjing Luo, Feng Xu, Xiaofeng Feng, Liangshun Zhang, Huiwei Liu, Jianwei Shen, Hua Ye

**Affiliations:** The Affiliated Lihuili Hospital of Ningbo University, Ningbo, Zhejiang, China

**Keywords:** NHANES, MAFLD, liver fibrosis, total folate, 5-MTHF

## Abstract

**Background:**

Metabolically Associated Fatty Liver Disease (MAFLD) marks a progression from the previous paradigm of Non-Alcoholic Fatty Liver Disease (NAFLD), presenting a redefined diagnostic framework that accentuates metabolic factors while recognizing non-alcoholic contributors. In our investigation, our principal aim was to scrutinize the conceivable correlation between diverse serum folate levels and the prevalence of MAFLD and liver fibrosis.

**Methods:**

In our investigation, we conducted an extensive analysis utilizing data derived from the National Health and Nutrition Examination Survey (NHANES) across the years 2017–2020. We aimed to investigate the association between different serum folate concentrations and the prevalence of MAFLD and liver fibrosis by comprehensive multivariate analysis. This analytical approach considered various variables, encompassing sociodemographic characteristics, lifestyle factors, hypertension, and diabetes. By including these potential confounders in our analysis, we aimed to ensure the stability of the findings regarding the association between different serum folate concentrations and the development of MAFLD and liver fibrosis.

**Results:**

In our investigation, we utilized multiple linear regression models to thoroughly analyze the data, revealing noteworthy insights. Evidently, elevated levels of both total folate and 5-MTHF exhibited a distinct negative correlation with CAP, while 5-MTHF demonstrated a notable negative correlation with LSM. Furthermore, multiple logistic regression models were employed for an in-depth examination of the data. As the concentrations of total folate and 5-MTHF in the serum increased, a substantial decrease in the likelihood of MAFLD and liver fibrosis occurrence was observed.

**Conclusion:**

The findings of this investigation robustly suggest the prevalence of MAFLD and liver fibrosis decreased significantly with the increase of serum concentrations of total folate and 5-MTHF.

## 1 Introduction

In 2020, a noteworthy paradigmatic transition transpired in the categorization of hepatic maladies with introduction of Metabolically Associated Fatty Liver Disease (MAFLD) (1, 2). This revolutionary framework augments the delineation of hepatic disorders by assimilating indicators of metabolic aberrations, encompassing insulin resistance, heightened susceptibility to C-reactive protein (Hs-CRP), and diverse other concomitant metabolic predisposing factors (1, 2). Deviating from Non-Alcoholic Fatty Liver Disease (NAFLD), which explicitly omits conditions such as viral hepatitis, alcoholic liver disease, and other hepatic disorders, the diagnostic criteria for MAFLD embrace a notably more pragmatic standpoint (3). Through the incorporation of metabolic markers, MAFLD endeavors to discern individuals exhibiting fatty liver conditions. These individuals not only meet the conventional criteria for NAFLD but also demonstrate elevated risks of disease progression (4). This conceptual progression underscores a nuanced comprehension of the intricate interplay between metabolic factors and hepatic well-being, ushering in a more comprehensive and clinically pertinent approach to the diagnosis and treatment of fatty liver disorders within the scientific milieu (4).

Folate, a water-soluble B9 vitamin crucial for one-carbon metabolism and methylation reactions exists in three main forms: 5-Methyltetrahydrofolate (5-MTHF), folic acid, and tetrahydrofolate (THF), each serving distinct functions and roles (5, 6). 5-MTHF, an active form used directly by the body, plays a crucial role in cellular processes like gene regulation, neurotransmitter synthesis, and detoxification (7). Folic acid, a synthetic form of folate found in supplements and fortified foods, needs to be converted into active forms like 5-MTHF in the body but is essential in prenatal supplements to prevent neural tube defects during pregnancy (8). THF is a middleman in the folate pathway, transporting one-carbon units crucial for creating nucleotides and amino acids. The body converts THF into different forms, including 5-MTHF, through enzymatic processes (9). Experimental studies have implicated folate in processes such as oxidative stress, hepatic lipid metabolism, and chronic inflammation in the liver, all recognized as risk factors for NAFLD (10). In addition, certain animal investigations have indicated that supplementing with folic acid may mitigate hepatic steatosis (11, 12). Furthermore, human studies have demonstrated that serum folate could potentially exert a significant role in averting or retarding the progression of Non-Alcoholic Steatohepatitis (NASH), along with the potential to reverse liver inflammation and fibrosis (13). Prior epidemiological analyses have also indicated an inverse association between higher concentrations of serum total folate, 5-MTHF and the prevalence of NAFLD, liver fibrosis (14).

To the best of our knowledge, prior investigations examining the association between various serum folate levels and NAFLD and liver fibrosis have utilized diverse analytical approaches. These analyses primarily relied on blood biomarker indicators, such as the United States Fatty Liver Index (USFLI), Fatty Liver Index (FLI), Fibrosis-4 score (FIB-4), and NAFLD Fibrosis Score (NFS) (14–16). Earlier studies have also explored the impact of serum total folate on MAFLD using Vibration-Controlled Transient Elastography (VCTE). However, these studies encompassed only a single cycle from 2017 to 2018, resulting in a relatively limited number of population samples (17). Our primary objective was to augment the sample size by incorporating participants who underwent VCTE from the 2017–2020 cycles of the National Health and Nutrition Examination Survey (NHANES). Subsequently, we delved into the exploration of cross-sectional associations between various serum folate levels and both MAFLD as well as liver fibrosis. The hypothesis underlying this investigation is that there may be a significant negative association between various serum folate levels and MAFLD, as well as liver fibrosis.

## 2 Materials and methods

### 2.1 Study population

**Figure 1:**
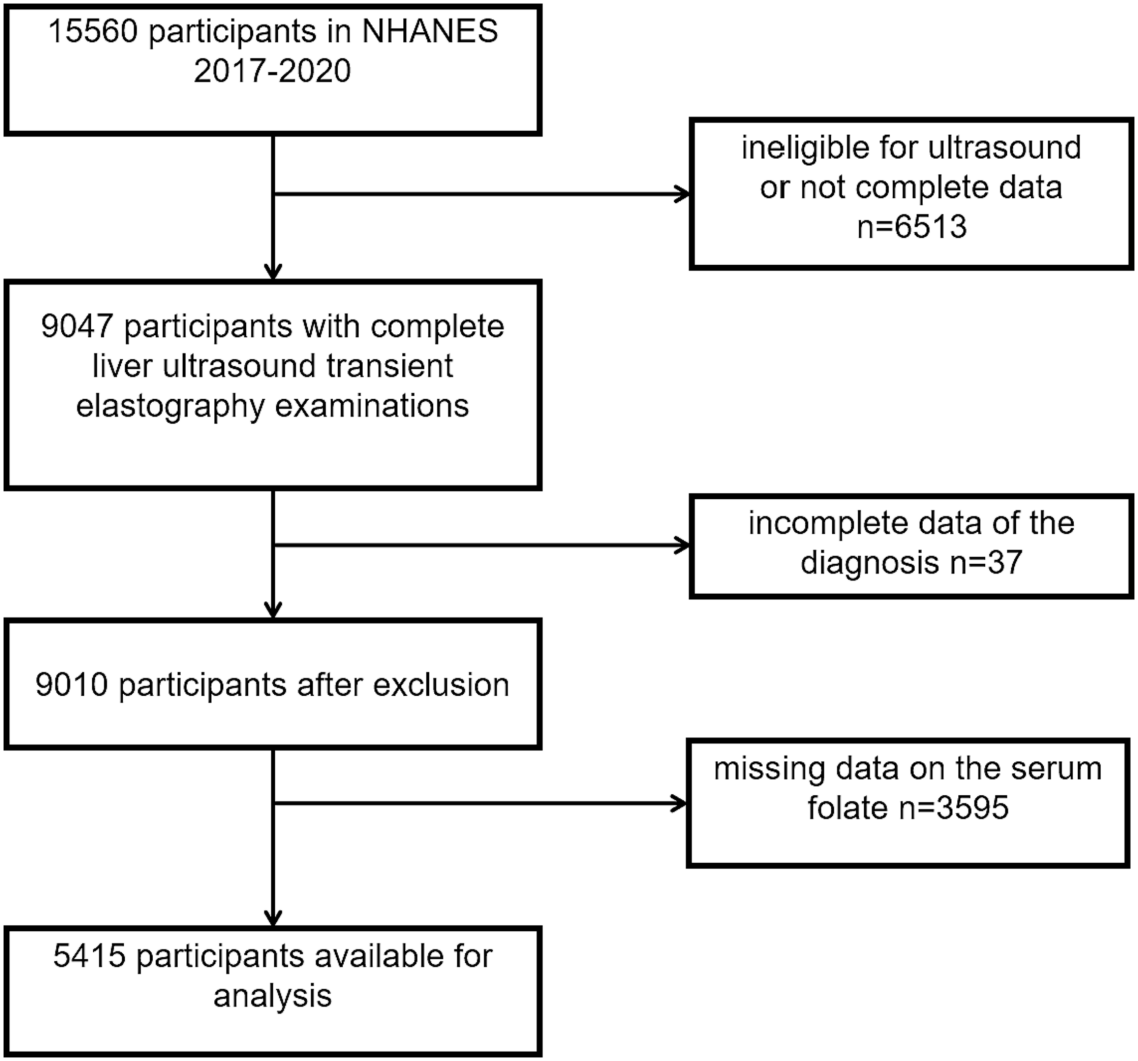
Flow chart of participants screening.

Conceived under the auspices of the National Center for Health Statistics (NCHS) and endorsed by the Centers for Disease Control and Prevention (CDC), NHANES stands as an intricately executed nationwide survey. Providing detailed insights into population characteristics, socioeconomic status, personal behaviors, individual health conditions, and pertinent health indicators derived from clinical laboratory assessments, NHANES serves as an invaluable instrument for acquiring nuanced insights into the multifaceted landscape of public health. The acquisition and dissemination of data within the NHANES database adhered to the Helsinki principles, with the requisite approval from the Ethics Committee to guarantee the ethical integrity of the data employed in this investigation. The dataset used in this investigation originates from the NHANES cycle spanning the years 2017–2020. From the initial cohort of 15,560 participants, several exclusions were implemented, outlined as follows: (1) individuals lacking comprehensive liver elastography measurements (*n* = 6,513), (2) those for whom the confirmation of MAFLD presence was inconclusive (*n* = 37), and (3) participants with incomplete data regarding serum folate (*n* = 3,595). Consequently, the definitive sample size was refined to 5,415 participants. For a more detailed representation, we refer the reader to Figure 1.

### 2.2 Measurement of various serum folate

For the analytical procedures, blood specimens were procured during the examination, subsequently frozen, and preserved at −30°C before being dispatched to CDC in Atlanta, Georgia. The quantification of five distinct folate forms (5-MTHF, folic acid, THF, 5-formyl-tetrahydrofolate, and 5,10-methenyl-tetrahydrofolate) was conducted using isotope-dilution high-performance liquid chromatography coupled to tandem mass spectrometry (LC–MS/MS; detailed procedures can be found at https://wwwn.cdc.gov/Nchs/Nhanes/2017-2018/P_FOLFMS.htm). As expounded on the NHANES website, in instances where the measured value falls below the lower limit of detection divided by the square root of 2 lower limits of detection (LLOD), a standard protocol was applied to impute the value by dividing the LLOD by the square root of 2. Due to a substantial number of measurements for 5-formyl-tetrahydrofolate and 5,10-methenyl-tetrahydrofolate falling below LLOD, these values were excluded from further analysis. Comprehensive information regarding the LLOD for various serum folate levels and the proportions above LLOD can be found in Supplementary Table 1. To facilitate the analysis, participants were stratified into three cohorts based on tertile concentrations of diverse serum folate levels.

### 2.3 Definition of MAFLD

The diagnosis of MAFLD was established when hepatic steatosis coexisted with any of the following criteria: being categorized as overweight or obese, indicated by a body mass index (BMI) ≥ 25, having diabetes mellitus, or manifesting signs of metabolic irregularities. These metabolic irregularities were specifically recognized when a patient exhibited at least two of the following metabolic risk abnormalities: (1) systolic/diastolic blood pressure surpassing 130/85 mmHg or the use of specific antihypertensive medications; (2) waist circumference (WC) ≥102 cm for males and ≥ 88 cm for females; (3) plasma high-density lipoprotein cholesterol (HDL) levels below 40 mg/dL for men and below 50 mg/dL for women, or the use of specific medications; (4) plasma triglyceride (TG) levels exceeding 150 mg/dL or the use of specific medications; (5) homeostasis model assessment of insulin resistance (HOMA-IR) score surpassing 2.5; (6) fasting plasma glucose (FPG) within the range of 5.6–6.9 mmol/L or Glycohemoglobin A1c (HbA1c) ranging from 5.7 to 6.4%; and (7) plasma Hs-CRP levels ≥2 mg/L (2).

### 2.4 Non-invasive assessment of hepatic steatosis and liver fibrosis

The assessment of hepatic steatosis at the NHANES Mobile Examination Center (MEC) involved employing elastography through VCTE. To maintain assessment accuracy, participants were obligated to observe a fasting period of no less than 3 h preceding the examination. Moreover, they were mandated to acquire more than 10 liver measurement values, ensuring an interquartile range/median ratio below 30% to bolster the reliability of the evaluation. Consistent with prior research endeavors, we defined liver steatosis as Controlled Attenuation Parameter (CAP) ≥ 238 dB/m (18, 19), while Liver Stiffness Measurement (LSM) ≥ 7 kPa indicated the presence of liver fibrosis (18).

### 2.5 Covariate assessment

The subsequent laboratory parameters encompassed WC, TG, total cholesterol (TC), low-density lipoprotein cholesterol (LDL), HDL, FPG, HbA1c, HOMA-IR, and Hs-CRP. Demographic data, including age, gender, race, BMI, educational attainment, marital status, poverty income ratio (PIR), alcohol consumption, smoking habits, and medication usage, were collected through standardized self-reported questionnaires. Educational achievements were categorized into three groups: “less than high school,” “high school or equivalent,” and “higher than high school.” Marital status was classified as “never married,” “married/cohabiting,” or “separated/divorced/widowed.” PIR was divided into three cohorts: <1.30, 1.30–3.50, and > 3.50 (20). Alcohol consumption was delineated into three groups: individuals who never consumed alcohol, moderate drinkers (1–2 drinks per day for males, 1 drink per day for females), and heavy drinkers (≥3 drinks per day for males, ≥2 drinks per day for females) (21). Smoking habits were categorized into three tiers: low (serum cotinine <0.015 ng/mL), moderate (0.015–3 ng/mL), and high level (serum cotinine >3 ng/mL) (22). According to the American Diabetes Association criteria, diabetes is characterized by the presence of any of the following criteria: FPG ≥ 126 mg/dL, HbA1c ≥ 6.5%, self-reported clinician-diagnosed diabetes, or initiation of drug treatment (23). Hypertension, as per the 2017 guidelines by the American Heart Association, is defined as a blood pressure reading surpassing 130/80 mm Hg or the initiation of specific drug treatment (24).

### 2.6 Statistical analysis

Continuous variables were expressed as mean values along with standard deviations (mean ± SD), while categorical variables were presented as percentages. The comparison of continuous variables utilized a weighted t-test, and for categorical variables, a chi-squared test was applied, with outcomes reported as counts (n) and percentages (%). Utilizing a linear regression model, we investigated the relationship between diverse serum folate levels and CAP as well as LSM. To assess the correlation between covariates and the analytical outcomes, three distinct models were formulated. Each model in the analysis progressively incorporated additional adjustments for covariates. The initial model remained unadjusted, while the second model included partial adjustments for age, race, gender, PIR, marital status, and educational level. Model 3 represents the fully adjusted model, encompassing additional variables such as BMI, smoking habits, alcohol consumption, diabetes, and hypertension. To assess the relationship between various serum folate levels and MAFLD as well as liver fibrosis, a multivariable logistic regression model was applied, utilizing the previously detailed three-level model. Following this, subgroup analyses were executed to explore potential modifications in effect measures, including gender, age, BMI, hypertension, and diabetes as potential influential factors. Moreover, a restricted cubic spline (RCS) analysis was performed to examine potential nonlinear associations between diverse serum folate levels and both MAFLD and liver fibrosis. All statistical analyses adhered to a significance level of less than 0.05 for a two-tailed *p* value. The software tools employed included STATA v16.0 (StataCorp LLC, College Station, TX, United States) and R (version 4.1.0, Vienna, Austria).

## 3 Results

### 3.1 Baseline characteristic

In our primary analysis, a total of 5,415 participants were included and Supplementary Table 2 furnishes further insights into the baseline attributes of these individuals. Comparative analysis between the non-MAFLD population and MAFLD patients revealed significant differences in gender, race, drinking habits, diabetes, hypertension, and BMI. Moreover, MAFLD patients exhibited higher levels of age, WC, TG, TC, LDL, FPG, Hb1Ac, HOMA-IR, and Hs-CRP, along with lower levels of HDL and 5-MTHF. Similar patterns were observed in patients with liver fibrosis compared to those without liver fibrosis, with notable distinctions in PIR, smoking habits, total folate, and THF. However, levels of TC and LDL did not show significant variations in the liver fibrosis group.

### 3.2 Association between various serum folate levels and CAP

The outcomes presented in Table 1 emanate from a series of multiple linear regression models aimed at exploring the potential association between concentrations of various serum folate levels and CAP. In Model 1, a noteworthy significant negative correlation was evident between serum total folate and 5-MTHF with CAP (*p* = 0.047, 0.015; respectively). Notably, Model 2 sustained a significant negative relationship between serum total folate and 5-MTHF with CAP (*p* = 0.002, <0.001; respectively). Lastly, in Model 3, the analysis consistently indicated a significant negative association between serum total folate and 5-MTHF with CAP (*p* = 0.016, 0.009; respectively). The outcomes of the linear regression analysis exploring the relationship between folic acid and THF with CAP are presented in Supplementary Table 3.

**Table 1:** Linear regression model between serum total folate, 5-MTHF, and CAP.

		Model 1		Model 2		Model 3	
		β (95% CI)	*p* trend	β (95% CI)	*p* trend	β (95% CI)	*p* trend
Total folate	Continuous	−0.099 (−0.197, −0.001)	0.047	−0.155 (−0.253, −0.057)	0.002	−0.097 (−0.175, −0.018)	0.016
	T1	ref	ref	ref	ref	ref	ref
	T2	−7.215 (−13.630, −0.800)	0.028	−4.734 (−10.919, 1.451)	0.134	−2.130 (−6.799, 2.539)	0.371
	T3	−8.901 (−15.124, −2.679)	0.005	−9.096 (−15.250, −2.943)	0.004	−5.393 (−10.233, −0.552)	0.029

5-MTHF	Continuous	−0.136 (−0.246, −0.026)	0.015	−0.203 (−0.312, −0.093)	<0.001	−0.117 (−0.205, −0.029)	0.009
	T1	ref	ref	ref	ref	ref	ref
	T2	−7.661(−14.092,-1.230)	0.020	−5.273(−11.468,0.922)	0.095	−2.469(−7.130,2.191)	0.299
	T3	−9.624(−15.839,-3.408)	0.002	−9.998(−16.172,-3.825)	0.002	−5.612(−10.447,-0.777)	0.023

Additionally, we conducted an analysis utilizing serum total folate and 5-MTHF concentrations as categorical variables. In Model 1, in comparison to the reference group (T1), elevated levels of serum total folate (T2 and T3 groups) and 5-MTHF (T2 and T3 groups) demonstrated a significant negative association with CAP (*p* all <0.05). Transitioning to Model 2, the highest serum total folate and 5-MTHF level exhibited a significant negative association with CAP (*p* = 0.004, 0.002; respectively). Analogously, in Model 3, the highest serum total folate and 5-MTHF concentrations continued to exhibit a significant negative association with CAP (*p* = 0.029, 0.023; respectively).

### 3.3 Association between various serum folate levels and LSM

The outcomes presented in Table 2 elucidate findings from a series of multiple linear regression models devised to explore the potential association between concentrations of various serum folate levels and LSM. In Model 1, a noteworthy significant negative correlation was observed between serum 5-MTHF and LSM (*p* = 0.031). Notably, Model 2 sustained a significant negative relationship between 5-MTHF and LSM (*p* = 0.011). Finally, in Model 3, the analysis consistently indicated a significant negative association between serum 5-MTHF and LSM (*p* = 0.019). However, in all three models, we did not find a significant correlation between serum total folate and LSM. The results of the linear regression analysis exploring the relationship between folic acid and THF with LSM are presented in Supplementary Table 4. Additionally, we conducted an analysis using serum total folate and 5-MTHF concentrations as categorical variables. Nonetheless, in this analysis, we did not identify a significant correlation between serum total folate, 5-MTHF concentrations, and LSM.

**Table 2:** Linear regression model between serum total folate, 5-MTHF, and LSM.

		Model 1		Model 2		Model 3	
		β (95% CI)	*p* trend	β (95% CI)	*p* trend	β (95% CI)	*p* trend
Total folate	Continuous	−0.002 (−0.009, 0.005)	0.589	−0.004 (−0.012, 0.004)	0.351	−0.003 (−0.010, 0.004)	0.406
	T1	ref	ref	ref	ref	ref	ref
	T2	0.072 (−0.389, 0.533)	0.761	0.117 (−0.352, 0.586)	0.625	0.051 (−0.420, 0.523)	0.831
	T3	−0.296 (−0.672, 0.080)	0.123	−0.307 (−0.711, 0.096)	0.135	−0.307 (−0.700, 0.086)	0.126

5-MTHF	Continuous	−0.006 (−0.011, −0.001)	0.031	−0.008 (−0.014, −0.002)	0.011	−0.007 (−0.013, −0.001)	0.019
	T1	ref	ref	ref	ref	ref	ref
	T2	−0.034 (−0.496, 0.429)	0.887	0.015 (−0.459, 0.489)	0.951	0.063 (−0.411, 0.537)	0.796
	T3	−0.363 (−0.749, 0.023)	0.065	−0.375 (−0.795, 0.046)	0.081	−0.303 (−0.697, 0.091)	0.132

### 3.4 Association between various serum folate levels and MAFLD

Table 3 encapsulates the outcomes derived from multiple logistic regression models scrutinizing the potential independent associations between serum total folate and 5-MTHF concentrations and MAFLD. A significant negative association between the highest level of serum total folate and 5-MTHF and MAFLD was observed in Model 1 (*p* = 0.008, 0.003; respectively). Model 2 revealed a significant negative association between the highest level of serum total folate and 5-MTHF (*p* = 0.025, 0.012; respectively) with MAFLD. According to Model 3, the likelihood of MAFLD decreased significantly as total folate and 5-MTHF (*p* = 0.042, 0.024; respectively) concentrations in the serumincreased. The outcomes of the logistic regression analysis examining the association of folic acid and THF with MAFLD are presented in Supplementary Table 5. We found a significant positive correlation between THF and MAFLD in Model 1 and Model 2 (*p* <0.001,= 0.006; respectively).

**Table 3:** Logistic regression model between serum total folate, 5-MTHF level, and MAFLD.

	MAFLD						
		Model 1		Model 2		Model 3	
		OR (95%CI)	*p* trend	OR (95%CI)	*p* trend	OR (95%CI)	*p* trend
Total Folate	T1	ref	ref	ref	ref	ref	ref
	T2	0.819 (0.651, 1.030)	0.088	0.929 (0.738, 1.170)	0.532	0.934 (0.723, 1.206)	0.600
	T3	0.736 (0.587, 0.923)	0.008	0.768 (0.609, 0.968)	0.025	0.758 (0.581, 0.990)	0.042

5-MTHF	T1	ref	ref	ref	ref	ref	ref
	T2	0.818 (0.651, 1.028)	0.085	0.870 (0.683, 1.108)	0.467	0.932 (0.722, 1.204)	0.590
	T3	0.706 (0.563, 0.886)	0.003	0.689 (0.536, 0.886)	0.012	0.736 (0.564, 0.961)	0.024

**Figure 2:**
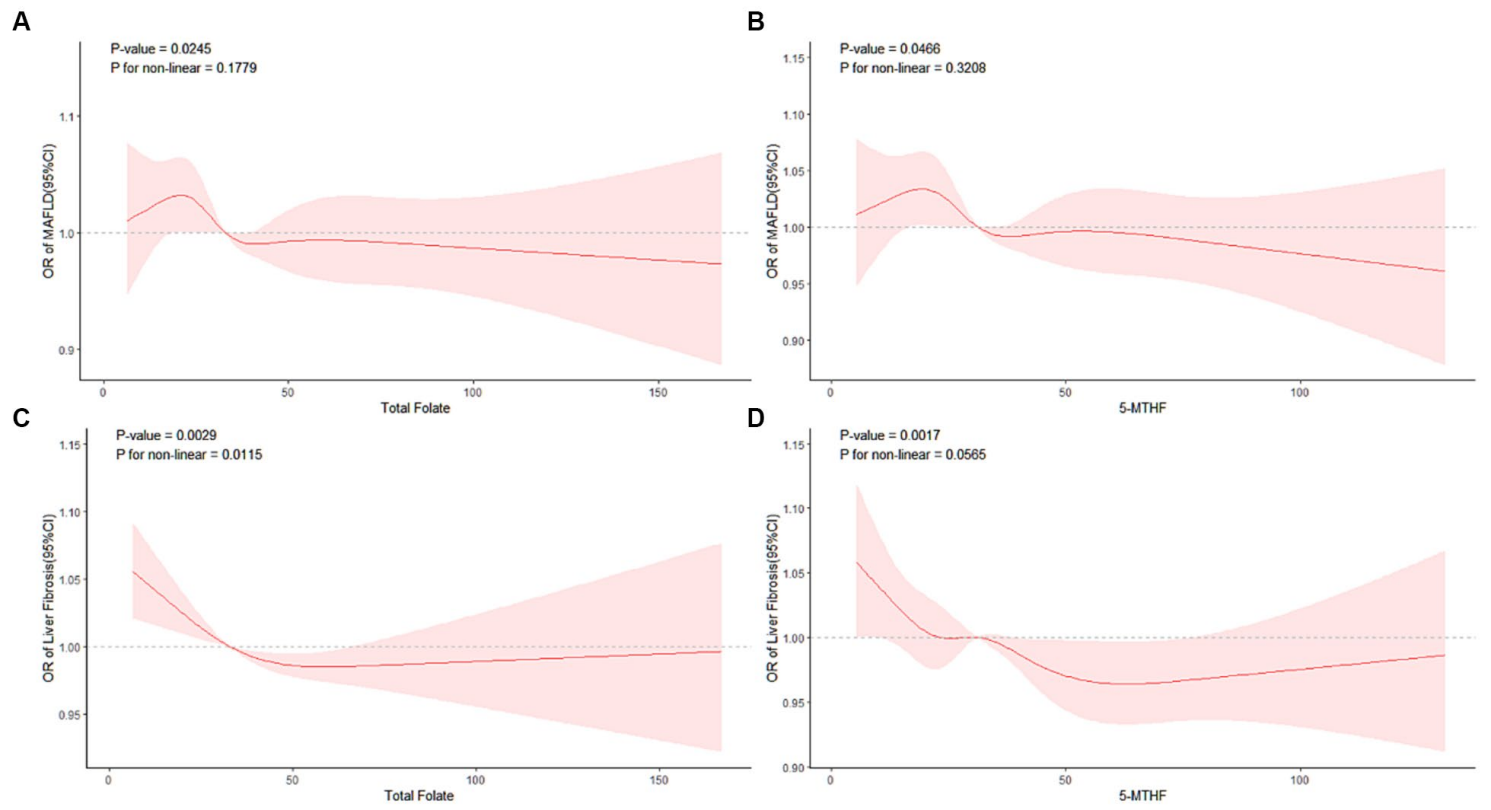
RCS analysis of the association of MAFLD with **(A)** total folate, **(B)** 5-MTHF, liver fibrosis with **(C)** total folate, and **(D)** 5-MTHF.

Following comprehensive multivariable adjustments in Model 3, no evident nonlinear relationship between serum total folate, 5-MTHF, and MAFLD was observed in RCS analysis (*p* overall = 0.0245, 0.0466; *p* nonlinear = 0.1779, 0.3208) (refer to Figures 2A,B).

### 3.5 Association between various serum folate levels and liver fibrosis

Table 4 presents the outcomes derived from multiple logistic regression models scrutinizing the potential independent associations between serum total folate and 5-MTHF concentrations and liver fibrosis. A significant negative association between blood levels of total folate (T2 and T3 group) and 5-MTHF (T2 and T3 group) and liver fibrosis was observed in Model 1 (*p* all<0.05). In Model 2, serum total folate levels (T3 group, *p* = 0.013) and 5-MTHF levels (T3 group, *p* = 0.006) were significantly negatively associated with liver fibrosis. Furthermore, in the final model, Model 3, the analysis demonstrated that as the concentration of serum total folate and 5-MTHF increased, the likelihood of liver fibrosis also decreased significantly (T3 group, *p* = 0.010, 0.005; respectively). The outcomes of the logistic regression analysis assessing the association of folic acid and THF with liver fibrosis are presented in Supplementary Table 6. A significant negative correlation between folic acid and liver fibrosis was identified solely in Model 3 (*p* = 0.014).

**Table 4:** Logistic regression model between serum total folate, 5-MTHF level, and liver fibrosis.

	Liver fibrosis						
		Model 1		Model 2		Model 3	
		OR (95%CI)	*p* trend	OR (95%CI)	*p* trend	OR (95%CI)	*p* trend
Total folate	T1	ref	ref	ref	ref	ref	ref
	T2	0.756 (0.587, 0.973)	0.030	0.868 (0.666, 1.133)	0.298	0.847 (0.641, 1.117)	0.239
	T3	0.722 (0.560, 0.932)	0.012	0.695 (0.522, 0.925)	0.013	0.677 (0.503, 0.910)	0.010

5-MTHF	T1	ref	ref	ref	ref	ref	ref
	T2	0.859 (0.659, 1.121)	0.263	0.848 (0.642, 1.118)	0.242	0.859 (0.659, 1.121)	0.263
	T3	0.666 (0.499, 0.888)	0.006	0.651 (0.483, 0.878)	0.005	0.666 (0.499, 0.888)	0.006

Additionally, we conducted a RCS analysis based on Model 3 to explore potential nonlinear relationships between serum total folate, 5-MTHF, and the occurrence of liver fibrosis. We found a significant nonlinear relationship between serum total folate and liver fibrosis(*p* overall = 0.0029; *p* nonlinear = 0.0115); meanwhile, no evident nonlinear relationship between serum 5-MTHF, and liver fibrosis was observed in the RCS analysis (*p* overall = 0.0017; *p* nonlinear = 0.0565) (refer to Figures 2C,D). A discernable reduction in the risk of developing liver fibrosis was noted with an elevation in total folate levels surpassing a critical inflection point, estimated at approximately 31.19 nmol/L. Beyond this threshold, total folate demonstrated a protective role against the progression of liver fibrosis, underscoring its potential importance in mitigating this hepatic pathology.

### 3.6 Subgroup analysis

**Figure 3:**
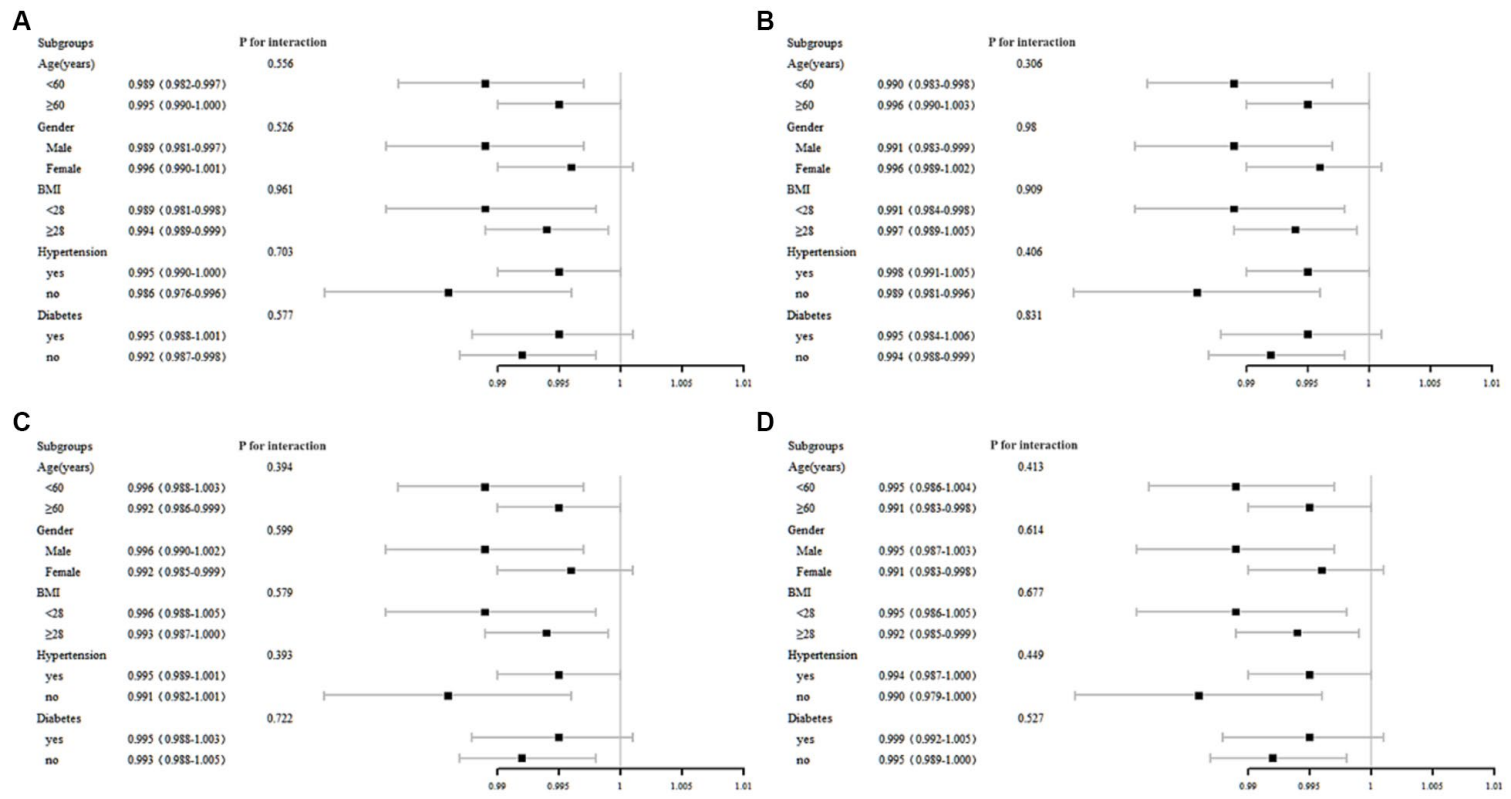
Subgroup analysis of the association between MAFLD with **(A)** total folate, **(B)** 5-MTHF, liver fibrosis with **(C)** total folate, and **(D)** 5-MTHF.

We performed stratified multivariate regression analysis to investigate the association between serum total folate, 5-MTHF, MAFLD, and liver fibrosis within distinct population subgroups categorized by gender (male/female), age (<60, ≥60), BMI (<28, ≥28), hypertension, and diabetes. As depicted in Figure 3, no significant significance was observed in all subgroup analyses of serum total folate and 5-MTHF with MAFLD and liver fibrosis (*p* all >0.05).

## 4 Discussion

Previous investigations have primarily focused on exploring the associations between serum total folate levels and the probability of NAFLD and liver fibrosis. However, to the best of our knowledge, there is a conspicuous dearth of epidemiological evidence pertaining to the potential correlations between various serum folate levels and MAFLD (15–17). Furthermore, prior investigations employing CAP and LSM to assess the impact of distinct serum folate types on hepatic steatosis and fibrosis were limited to a single survey cycle, resulting in a smaller study population (25). To address these knowledge gaps, we conducted an expansive study involving participants in the United States, leveraging data obtained from NHANES cycles spanning from 2017 to 2020 to encompass a more extensive cohort. This investigation exhibits distinctiveness through methodological advancements. In contrast to antecedent studies, which predominantly scrutinized predictors without employing VCTE, our approach integrates the latest VCTE data, thereby elevating the precision of our analysis (14–16). Furthermore, diverging from earlier inquiries focused on MAFLD that solely considered serum total folate levels from a single cycle (17), our study spans the period from 2017 to 2020. This allows us to not only examine the correlation between diverse serum folate levels and MAFLD but also results in a substantial augmentation of the sample size. This enlargement contributes to a more robust and generalizable investigation, facilitating a more accurate comprehension of the relationship between various serum folate levels and the occurrence of MAFLD, along with its implications for liver fibrosis. Throughout our inquiry, we discerned a noteworthy and persistent inverse correlation between serum total folate, 5-MTHF, and MAFLD, liver fibrosis. These observations align with antecedent investigations in the domain and retain their robustness even following adjustments for diverse potential confounding factors, encompassing sociodemographic characteristics, lifestyle elements, hypertension, and diabetes (14, 25). Nevertheless, our subgroup analyses revealed no significant distinctions in variables, a finding that aligns with previous studies (14, 16).

Research has substantiated the potential of folate to ameliorate MAFLD through diverse pathways. Primarily, folate assumes a crucial role in purine and thymidylate synthesis as well as DNA methylation. Folate deficiency, in turn, is implicated in the heightened expression of genes associated with hepatic fat synthesis, consequently leading to the development of hepatic steatosis (26). Furthermore, inadequate folate levels may disrupt the fibroblast growth factor (FGF) pathways. FGF concentrations have been correlated with insulin resistance, and the regulation of visceral adiposity, by FGF stimulates adipokines and inflammatory factors, thereby fostering the progression of lipotoxic liver disease (27, 28). Thirdly, folate exerts regulatory control over the transcription of NADPH oxidase, effectively mitigating the oxidative stress induced by a high-fat diet (29). This action results in increased adenosine monophosphate (AMP) levels and the activation of liver kinase B1 (LKB1), subsequently restoring adenosine monophosphate-activated protein kinase (AMPK) activation in the liver. Consequently, this regulatory mechanism contributes to the enhancement of cholesterol and glucose metabolism (30). Fourthly, folate, through the modulation of hepatic microRNA expression, has the potential to reduce blood glucose and lipid concentrations, enhance insulin sensitivity, and ameliorate liver function (31). These findings align with our study, indicating a correlation between elevated serum concentrations of both total folate, 5-MTHF and a decreased prevalence of MAFLD.

Our investigation revealed no substantial correlation between the presence of folic acid, THF and the prevalence of MAFLD. Similarly, another study focusing on young adults did not identify a significant association between folic acid and MAFLD (25). In contrast, Yang et al. (14) reported a significant positive correlation between serum folic acid concentration and the prevalence of NAFLD in their study. Elevated serum folic acid levels were linked to an increase in pro-inflammatory cytokines (IL-8 and TNF-α) and a decrease in natural killer cell cytotoxicity, potentially contributing to the onset of NAFLD (32, 33). Notably, the definition of NAFLD varied between studies, with Yang et al. relying on the USFLI or the FLI, while our study and Wen et al. defined NAFLD/MAFLD using CAP obtained through VCTE. This discrepancy in defining NAFLD/MAFLD may account for the observed inconsistencies in results. Specifically, our study identified a significant inverse association between THF and MAFLD only in Model 1 and 2. In preceding murine investigations, enhancements in intestinal microbiota have demonstrated a concurrent mitigation of steatosis and elevation in hepatic THF content (34). Nonetheless, a paucity of precise experimental or epidemiological inquiries has been undertaken to elucidate the association between THF and hepatic steatosis. Our present study did not reveal a noteworthy correlation between folic acid, THF, and MAFLD. Our hypothesis posits that the underlying cause may be attributed to the comparatively limited proportion of folic acid and THF, constituting a minor fraction of total folate, typically accounting for less than 5% on average. Further studies are warranted to validate our findings, and additional investigations into potential underlying mechanisms are essential.

Beyond its demonstrated efficacy in mitigating liver steatosis, investigations have substantiated the potential of folate to ameliorate liver fibrosis through diverse pathways. A pivotal mediator in hepatocellular injury is hepatic oxidative stress. Folate, with its antioxidant attributes, has been proposed to play a crucial role by directly scavenging Reactive Oxygen Species (ROS), enhancing hepatic lipid peroxidation, and impeding the activities of various antioxidant enzymes (10). Moreover, an investigation has revealed that folate supplementation induces an elevation in the hepatic expression of the fusion protein Syntaxin 17, a crucial factor in counteracting the advancement of liver inflammation and fibrosis (13). The one-carbon units linked to folates serve dual roles—they can undergo oxidation, contribute to the remethylation of homocysteine to generate methionine, or act as substrates for *de novo* purine and thymidine synthesis. Consequently, folate deficiency may disrupt *de novo* phosphatidylcholine synthesis and perturb purine signaling, hastening the progression of liver fibrosis (5). The liberation of inflammatory cytokines orchestrated by macrophages is implicated in the progression of vascular disease. Folate has been demonstrated to mitigate homocysteine-induced pro-inflammatory cytokine expression and impede the recruitment and activation of Kupffer cells, consequently diminishing the likelihood of liver fibrosis (10). In our investigation, a significant negative association between serum total folate and 5-MTHF concentration and the occurrence of liver fibrosis was observed, except THF. While our study revealed a substantial negative correlation between folic acid and liver fibrosis in Model 3, this association was not evident in Models 1 and 2, or in the linear regression analyses. Therefore, we cautiously conclude that the current evidence for this relationship is not highly feasible. Further research examining the impact of various serum folate forms on liver fibrosis is warranted for a more comprehensive understanding of these relationships.

This study marks the inaugural investigation into the correlation between diverse serum folate levels and MAFLD. Notably, it encompasses the largest NHANES population with CAP and LSM data within the 2017–2020 cycle. These findings underscore the importance of elevating public awareness regarding the influence of heightened serum folate concentrations on MAFLD and liver fibrosis. Public health initiatives can amplify awareness by emphasizing the impact of serum folate on metabolic health, targeting the general population. These campaigns should underscore the necessity of maintaining optimal folate levels. In clinical practice, healthcare providers should personalize treatments for individuals with metabolic disorders, recommending folate-rich foods or medications to mitigate associated risks. Moreover, this study prompts consideration for future research directions on serum folate in MAFLD and liver fibrosis. Potential investigations include exploring the dose–response correlation between folate intake and MAFLD as well as liver fibrosis, discerning the impact of diverse folate levels on outcomes. Additionally, evaluating the feasibility and efficacy of incorporating routine folate into clinical practice. This may enhance the precision of dietary or supplemental recommendations, contributing to more personalized and effective strategies for managing metabolic health.

Employing a comprehensive approach that integrates multifactor regression analysis and subgroup analysis, we assessed the influence of various serum folate levels on MAFLD and liver fibrosis. Despite these strengths, certain limitations merit consideration. The cross-sectional design of the NHANES dataset inherently precludes the establishment of causal relationships. Despite meticulous covariate control, the potential influence of unaccounted variables on the association between diverse serum folate levels and MAFLD, liver fibrosis remains a consideration. Genetic factors, specific dietary patterns, or unaccounted lifestyle habits not addressed in the study may serve as confounding variables. For example, variations in participants’ exercise habits related to liver conditions could introduce biases, thereby complicating the accurate attribution of observed effects solely to serum folate levels. Additionally, the absence of standardized diagnostic thresholds for liver steatosis and fibrosis through VCTE poses a significant challenge. Researchers often employ diverse criteria, rendering cross-study comparisons challenging. Consequently, the precision of estimates may be compromised. Moreover, the study recognizes the impact of variability in diagnostic criteria for comorbid conditions, such as diabetes, alcohol consumption, and cotinine exposure. The adoption of diverse criteria across studies results in heterogeneous participant categorization, creating challenges in drawing uniform conclusions. For example, our study uses cotinine levels, while other employs self-reported smoking numbers. These methodological differences may introduce discrepancies that could affect the accuracy of assessing the association between serum folate levels and liver health. Finally, concerns surrounding MAFLD have emerged due to its heterogeneous etiologies, and the term “fatty” has faced criticism for its potential stigmatizing effects (35). In 2023, an international panel of experts recommended transitioning from MAFLD to metabolic dysfunction-associated steatotic liver disease (MASLD). The updated diagnostic criteria for MASLD require the fulfillment of at least one of the five cardiometabolic risk factors, contingent upon the presence of liver steatosis (36). MAFLD’s limitations stem from evolving diagnostic criteria, possibly leading to heterogeneity in study populations.

## 5 Conclusion

In this expansive cross-sectional investigation encompassing a substantial population, we have established a significant negative correlation between heightened serum concentrations of total folate, 5-MTHF, and CAP. Simultaneously, elevated serum 5-MTHF levels are significantly associated with lower LSM. Furthermore, we have discerned a significant relationship between increased serum concentrations of both total folate and 5-MTHF and a decreased prevalence of MAFLD and liver fibrosis. Our findings have undergone meticulous adjustments for various potential confounders, reaffirming the robustness of these associations. These results underscore the significance of enhancing public awareness regarding the impact of elevated serum folate concentrations on metabolic health.

## Data availability statement

The data utilized in this investigation were sourced from NHANES, and detailed information can be accessed at: https://wwwn.cdc.gov/nchs/nhanes/Default.aspx.

## Ethics statement

The studies involving humans were approved by Ethical approval for the study was granted by the Research Ethics Review Board of the National Center for Health Statistics. The studies were conducted in accordance with the local legislation and institutional requirements. Written informed consent for participation in this study was provided by the participants’ legal guardians/next of kin.

## Author contributions

JC: Conceptualization, Data curation, Formal Analysis, Methodology, Software, Writing – original draft, Writing – review & editing. DC: Software, Supervision, Writing – review & editing. WL: Writing – review & editing. FX: Writing – review & editing. XF: Writing – review & editing. LZ: Writing – review & editing. HL: Writing – review & editing. JS: Funding acquisition, Project administration, Supervision, Writing – review & editing. HY: Funding acquisition, Investigation, Project administration, Resources, Supervision, Writing – review & editing.
