# Supplementary material for: The association between diverse serum folate with MAFLD and liver fibrosis based on NHANES 2017–2020
Source: Front Nutr. 2024 Mar 19;11:1366843. doi: 10.3389/fnut.2024.1366843 (PMC10986760; doi:10.3389/fnut.2024.1366843)
Supplement: Supplementary file 2 [file Table_1.docx]

**Supplementary table1: Detection frequency of various serum folate in NHANES**

| various serum folate | LLOD(nmol/L) | proportion above the LLOD(%) |
| --- | --- | --- |
| 5-MTHF | 0.13 | 100 |
| folic acid | 0.14 | 99.75 |
| THF | 0.25 | 83.85 |

**Supplementary table2: Characteristics of participants included**

|  |  | Non-MAFLD | MAFLD | P-value | Non- liver fibrosis | Liver fibrosis | P-value |
| --- | --- | --- | --- | --- | --- | --- | --- |
| Age (year) | | 37.72(0.61) | 47.88(0.48) | <0.001 | 42.56(0.61) | 48.74(1.20) | <0.001 |
| Gender(n,%) | | | | <0.001 |  | | <0.001 |
| Male | | 802(34.08) | 1,336(43.63) |  | 1,642(37.09) | 496(50.20) |  |
| Female | | 1,551(65.92) | 1,726(56.37) |  | 2,785(62.91) | 492(49.80) |  |
| Race(n,%) | | | | <0.001 |  | | <0.001 |
| Mexican American | | 244(10.37) | 490(16.00) |  | 589(13.71) | 145(12.97) |  |
| Other Hispanic | | 233(9.90) | 312(10.19) |  | 440(10.24) | 105(9.39) |  |
| Non-Hispanic white | | 764(32.47) | 1,043(34.06) |  | 1,416(32.95) | 391(34.97) |  |
| Non-Hispanic black | | 672(28.56) | 708(23.12) |  | 1,053(24.51 ) | 327(29.25) |  |
| Other | | 440(18.70) | 509(16.62) |  | 799(18.59) | 150(13.42) |  |
| Education level(n,%) | | | | 0.576 |  | | 0.356 |
| Less than high school | | 468(19.89) | 589(19.23) |  | 895(20.22) | 202(20.44) |  |
| High school or equivalent | | 571(24.27) | 746(24.36) |  | 1,090(24.62) | 267(27.02) |  |
| Above high school | | 1,314(55.84) | 1,727(56.41) |  | 2,442(55.16) | 519(52.54) |  |
| Marital status (n, %) | | | | 0.672 |  | | 0.103 |
| Married/cohabitant | | 1,618(68.76) | 2,005(65.48) |  | 3,011(68.01) | 612(61.94) |  |
| Widowed/divorced/separated | | 299(12.71) | 574(18.75) |  | 653(14.75) | 220(22.27) |  |
| Never married | | 436(18.53) | 483(15.77) |  | 763(17.24) | 156(15.79) |  |
| Poverty income ratio (n, %) | | | | 0.131 |  | | 0.001 |
| <1.30 | | 671(28.52) | 795(25.96) |  | 1,171(26.45) | 295(29.86) |  |
| 1.30-3.50 | | 747(31.75) | 1,041(34.00) |  | 1,432(32.35) | 356(36.03) |  |
| >3.50 | | 935(39.73) | 1,226(40.04) |  | 1,824(41.20) | 337(34.11) |  |
| Drinking status (n, %) | | | | <0.001 |  | | 0.002 |
| Non | | 1,117(47.47) | 1,103(36.02) |  | 1,859(41.99) | 361(36.54) |  |
| Moderate | | 547(23.25) | 965(31.52) |  | 1,199(27.08) | 313(31.68) |  |
| Heavy | | 689(29.28) | 994(32.46) |  | 1,369(30.92) | 314(31.78) |  |
| Smoking status (n, %) | | | | 0.324 |  | | 0.006 |
| Low level | | 769(32.68) | 1,064(34.75) |  | 1,533(34.63) | 300(30.36) |  |
| Moderate level | | 997(42.37) | 1,329(43.40) |  | 1,892(42.74) | 434(43.93) |  |
| High level | | 587(24.95) | 669(21.85) |  | 1,002(22.63) | 254(25.71) |  |
| Diabetes (n, %) | | | | <0.001 |  | | <0.001 |
| Yes | | 299(7.84) | 659(21.52) |  | 487(11.00) | 287(29.05) |  |
| No | | 2,238(95.11) | 2,403(78.48) |  | 3,940(89.00) | 701(70.95) |  |
| Hypertension(n, %) | | | | <0.001 |  | | <0.001 |
| Yes | | 662(28.13) | 1,679(54.83) |  | 1,727(39.01) | 614(62.15) |  |
| No | | 1,691(71.87) | 1,383(45.17) |  | 2,700(60.99) | 62.15(37.85) |  |
| BMI (kg/m2) | | | | <0.001 |  | | <0.001 |
| <28 | | 1,835(77.99) | 964(31.48) |  | 2,493(56.31) | 306(30.97) |  |
| ≥28 | | 518(22.01) | 2,098(68.52) |  | 1,934(43.69) | 682(69.03) |  |
| WC(cm) | | 86.29(0.39) | 105.93(0.44) | <0.001 | 95.54(0.36) | 109.88(0.99) | <0.001 |
| TG (mg/dL) | | 74.90(1.79) | 126.31(4.17) | <0.001 | 102.86(2.98) | 122.56(3.49) | <0.001 |
| TC (mg/dL) | | 174.37(1.27) | 187.82(1.19) | <0.001 | 183.20(1.02) | 180.00(1.81) | 0.546 |
| LDL (mg/dL) | | 101.04(1.72) | 111.79(1.52) | <0.001 | 108.29(1.32) | 104.68(1.51) | 0.341 |
| HDL (mg/dL) | | 57.89(0.51) | 50.44(0.39) | <0.001 | 54.07(0.36) | 50.27(0.70) | <0.001 |
| FPG(mmol/L)) | | 5.54(0.03) | 6.34(0.08) | <0.001 | 5.90(0.05) | 6.62(0.14) | <0.001 |
| Hb1Ac(%) | | 5.37(0.01) | 5.77(0.03) | <0.001 | 5.52(0.02) | 5.97(0.05) | <0.001 |
| HOMA-IR | | 2.16(0.11) | 5.41(0.47) | <0.001 | 3.21(0.14) | 8.11(0.45) | <0.001 |
| Hs-CRP(mg/L) | | 2.55(0.18) | 3.94(0.18) | <0.001 | 3.25(0.13) | 4.52(0.28) | <0.001 |
| Total folate(nmol/L) | | 41.26(0.80) | 39.47(0.66) | 0.072 | 40.63(0.58) | 38.21(1.03) | 0.019 |
| 5-MTHF(ug/L) | | 38.82(0.74) | 36.86(0.60) | 0.008 | 38.11(0.54) | 35.59(0.93) | 0.001 |
| Folic acid(ug/L) | | 1.43(0.14) | 1.53(0.15) | 0.213 | 1.48 (0.11) | 1.54(0.29) | 0.070 |
| THF(ug/L) | | 0.71(0.02) | 0.77(0.01) | 0.050 | 0.74(0.01) | 0.79(0.02) | <0.001 |

**Supplementary table3: Linear regression model between serum folic acid, THF and CAP**

|  |  | CAP | | | | | | | |
| --- | --- | --- | --- | --- | --- | --- | --- | --- | --- |
|  |  | model1 | | model2 | | | | model3 | |
|  |  | β, (95% CI) | P trend | β, (95% CI) | | P trend | | β, (95% CI) | P trend |
| Folic acid | Continuous | 0.064(-0.228,0.356) | 0.669 | 0.041(-0.218,0.300) | | 0.758 | | -0.097(-0.299,0.186) | 0.649 |
|  | T1 | ref | ref | ref | | ref | | ref | ref |
|  | T2 | -1.986(-8.356,4.384) | 0.541 | 0.041(-5.994,6.076) | | 0.989 | | 0.006(-4.706,4.719) | 0.998 |
|  | T3 | -4.212(-10.389,1.965) | 0.181 | -3.322(-9.396,2.752) | | 0.284 | | -4.252(-8.872,0.368) | 0.071 |
|  | | | | | | | | | |
| THF | Continuous | 8.074(3.110,13.037) | 0.001 | 3.599(-0.909,8.107) | | 0.118 | | -0.286(-3.771,3.198) | 0.872 |
|  | T1 | ref | ref | ref | | ref | | ref | ref |
|  | T2 | -7.661(-14.092,-1.230) | 0.020 | | 2.867(-3.130,8.863) | 0.349 | -1.501(-6.135,3.133) | | 0.525 |
|  | T3 | -9.624(-15.839,-3.408) | <0.001 | 11.108(5.261,16.955) | | <0.001 | | 3.140(-1.379,7.659) | 0.173 |

**Supplementary table4: Linear regression model between serum folic acid, THF and LSM**

|  |  | LSM | | | | | |
| --- | --- | --- | --- | --- | --- | --- | --- |
|  |  | model1 | | model2 | | model3 | |
|  |  | β, (95% CI) | P trend | β, (95% CI) | P trend | β, (95% CI) | P trend |
| Folic acid | Continuous | 0.033(-0.007,0.072) | 0.103 | 0.030(-0.008,0.069) | 0.121 | 0.026(-0.011,0.063) | 0.165 |
|  | T1 | ref | ref | ref | ref | ref | ref |
|  | T2 | 0.112(-0.324,0.547) | 0.615 | -0.020(-0.468,0.429) | 0.931 | -0.053(-0.495,0.389) | 0.814 |
|  | T3 | 0.525(0.091,0.959) | 0.018 | -0.070(-0.488,0.347) | 0.741 | -0.116(-0.537,0.305) | 0.590 |
|  | | | | | | | |
| THF | Continuous | 0.563(0.108,1.018) | 0.015 | 0.433(-0.042,0.908) | 0.074 | 0.309(-0.134,0.753) | 0.171 |
|  | T1 | ref | ref | ref | ref | ref | ref |
|  | T2 | -0.010(-0.460,0.439) | 0.964 | 0.073(-0.350,0.496) | 0.736 | -0.034(-0.466,0.398) | 0.878 |
|  | T3 | -0.007(-0.429,0.414) | 0.973 | 0.364(-0.068,0.796) | 0.099 | 0.159(-0.282,0.600) | 0.480 |

**Supplementary table5: Logistic regression model between serum folic acid, THF level and MAFLD**

|  | MAFLD | | | | | | |
| --- | --- | --- | --- | --- | --- | --- | --- |
|  |  | model1 | | model2 | | model3 | |
|  |  | OR (95%CI) | P trend | OR (95%CI) | P trend | OR (95%CI) | P trend |
| Folic acid | T1 | ref | ref | ref | ref | ref | ref |
|  | T2 | 0.980(0.781,1.229) | 0.860 | 0.994(0.801,1.233) | 0.955 | 1.016(0.793,1.302) | 0.901 |
|  | T3 | 0.851(0.680,1.064) | 0.157 | 0.905(0.714,1.148) | 0.412 | 0.873(0.668,1.142) | 0.321 |
|  | | | | | | | |
| THF | T1 | ref | ref | ref | ref | ref | ref |
|  | T2 | 1.170(0.919,1.488) | 0.203 | 1.095(0.875,1.370) | 0.430 | 0.947(0.736,1.217) | 0.669 |
|  | T3 | 1.570(1.251,1.971) | <0.001 | 1.374(1.098,1.719) | 0.006 | 1.067(0.824,1.383) | 0.621 |

**Supplementary table6: Logistic regression model between serum folic acid, THF level and liver fibrosis**

|  | Liver fibrosis | | | | | | |
| --- | --- | --- | --- | --- | --- | --- | --- |
|  |  | model1 | | model2 | | model3 | |
|  |  | OR (95%CI) | P trend | OR (95%CI) | P trend | OR (95%CI) | P trend |
| Folic acid | T1 | ref | ref | ref | ref | ref | ref |
|  | T2 | 0.927(0.719,1.195) | 0.557 | 0.624(0.691,1.078) | 0.155 | 0.804(0.609,1.061) | 0.124 |
|  | T3 | 0.864(0.669,1.115) | 0.261 | 0.727(0.552,0.957) | 0.086 | 0.694(0.519,0.928) | 0.014 |
|  | | | | | | | |
| THF | T1 | ref | ref | ref | ref | ref | ref |
|  | T2 | 0.957(0.731,1.254) | 0.751 | 0.966(0.728,1.283) | 0.812 | 0.905(0.672,1.217) | 0.508 |
|  | T3 | 1.226(0.954,1.575) | 0.112 | 1.061(0.807,1.396) | 0.671 | 0.901(0.677,1.200) | 0.476 |
